# Prognostic value of pretreatment FDG PET/CT in uterine cervical cancer according to two major histologic types: squamous cell carcinoma and adenocarcinoma

**DOI:** 10.22038/AOJNMB.2023.69159.1481

**Published:** 2023

**Authors:** Tomoaki Otani, Kanae Kawai Miyake, Takayoshi Ishimori, Aki Kido, Tsuneo Saga, Yuji Nakamoto

**Affiliations:** 1 Department of Diagnostic Imaging and Nuclear Medicine, Graduate School of Medicine, Kyoto University, Japan; 2 Department of Diagnostic Radiology, Japanese Red Cross Society Wakayama Medical Center, Japan; 3 Department of Diagnostic Radiology, Tazuke Kofukai Medical Research Institute, Kitano Hospital, Japan

**Keywords:** Cervical uterine cancer, Uterine cervical, adenocarcinoma, Prognostic value, FDG

## Abstract

**Objective(s):**

The aim of this study was to assess the prognostic value of pretreatment Positron emission tomography / computed tomography using ^18^F-fluorodeoxyglucose (FDG-PET/CT) in cervical cancer according to two major histologic types.

**Methods:**

Eighty-three squamous cell carcinoma (SCC) patients and 35 adenocarcinoma (AC) patients who underwent pretreatment FDG-PET/CT were retrospectively analyzed. Maximum standardized uptake value (SUV_max_), mean standardized uptake value (SUV_mean_), metabolic tumor volume (MTV), and total lesion glycolysis (TLG) of the primary tumor were calculated. Kaplan-Meier analyses were used to compare correlations between each PET parameter and overall survival (OS). The prognostic values of imaging and clinical parameters were assessed using uni- and multivariable Cox proportional hazard models.

**Results:**

SUV_max_, SUV_mean_, and TLG were significantly higher in SCC than in AC (p<0.01 each). No significant difference in MTV was seen between the two groups (p=0.10). As for Kaplan-Meier analyses, in SCC, patients with SUV_max_, SUV_mean_, MTV, and TLG exceeding cutoff values tended to show worse OS than patients with lower values (p=0.07, p=0.27, p<0.01, and p=0.01, respectively, for OS). On the other hand, in AC, patients with MTV and TLG exceeding cutoff values showed significantly worse PFS and OS (p<0.01 each for OS), while SUV_max_ and SUV_mean_ were unrelated to OS (p=0.91 and p=0.83, respectively for OS). As for multivariable analyses, in SCC, TLG was identified as an independent prognostic factor for OS (p=0.01). In AC, MTV was identified as an independent prognostic factor for OS (p=0.02).

**Conclusion:**

Our preliminary data suggest that FDG-PET/CT would be useful for predicting prognosis in cervical cancer, although the clinical significance of quantitative values may differ according to histopathological type.

## Introduction

 Uterine cervical cancer is one of the most common cancers affecting women worldwide (1). Two major histopathological types are seen in uterine cervical cancer: squamous cell carcinoma (SCC) and adenocarcinoma (AC) (2). SCC is the most common form, accounting for approximately 75% of all cervical cancers.

 Although AC is the second common, comprising 10–25% of all cervical carcinomas (3), the incidence is increasing (4, 5). Many differences exist between SCC and AC, including rates of metastasis, sites of recurrence, and response to chemotherapy (3, 6), so they are dealt with separately in clinical research. Several studies have indicated that AC is associated with poorer prognosis than SCC in both early and advanced stages (7, 8), but other studies, mostly focusing on early-stage disease, have failed to identify significant differences (9, 10). Whether histopathological type, as SCC or AC, represents an independent prognostic factor thus remains controversial.

 Positron emission tomography (PET)/ computed tomography (CT) using ^18^F-fluorodeoxyglucose (FDG) is a valuable tool in the management of patients with cervical cancer in initial staging, radiotherapy planning, response assessment, and re-staging of recurrence. Various quantitative PET/CT indices, including maximum standardized uptake value (SUV_max_), metabolic tumor volume (MTV), and total lesion glycolysis (TLG) of primary lesions, have been found to be useful predictors of survival in patients with cervical cancer (11-17). However, most previous studies have been performed by mixing SCC and AC in a single patient population. Rahman et al and Chou et al has investigated the prognostic value of PET/CT in cervical cancer patients according to histopathological type (18, 19), however, these studies dealt with AC and adenosquamous carcinoma (ASC) as a same group. To the best of our knowledge, no study examined the prognosis of purely AC group using PET/CT until now.

 The aim of this retrospective study was to investigate the prognostic value of pre-treatment PET/CT in cervical cancer, according to histopathological types of SCC and AC.

## Methods

***Patient characteristics and treatment***

 This study received approval from the ethics committee at our institute. Because of the anonymous nature of the data and the retrospective study design, the requirement for written informed consent was waived. We evaluated consecutive patients with cervical cancer (FIGO stages IB–IVA) receiving both FDG-PET/CT and MRI as a part of staging prior to treatment between May 2009 and August 2019. Minor histopathological types other than SCC and AC were excluded. In total, 118 patients (SCC, n=83; AC, n=35) were enrolled. Clinical staging was determined by physical examination performed by gynecologic oncologists according to FIGO 2018 staging criteria (20, 21). Histological diagnosis was determined by cervical biopsy determined by board-certified pathologists.

 The characteristics of this study population including age, FIGO stage, and treatment method are listed in Table 1. For the histological subtype of AC, 26 patients (74%) were classified with usual type, 5 patients (14%) with gastric-type mucinous AC (GAC), 1 patient (3%) with mucinous AC signet ring cell type, and 3 patients (9%) with poorly differentiated. AC patients were significantly younger than SCC patients (p=0.01).

**Table 1:** Characteristics of the study population

	**SCC (n=83)**	**AC (n=35)**
**Age**	24-95 (57)	22-80 (48)
**FIGO**		
ⅠB	25 (30%)	15 (43%)
Ⅱ	22 (27%)	6 (17%)
Ⅲ	31 (37%)	11 (31%)
Ⅳ	5 (6%)	3 (9%)
**Treatment method**		
Surgery	49 (59 %)	28 (80%)
Concurrent chemoradiotherapy (CCRT)	29 (35 %)	4 (11%)
Only radiation	5 (6 %)	1 (3%)
Only chemotherapy	0 (0%)	2 (6%)

 As for treatment method, 49 SCC patients and 28 AC patients underwent surgery. Among these, 46 SCC patients and 28 AC patients underwent radical hysterectomy, bilateral salpingo-oophorectomy and pelvic lymph-adenectomy. In 15 SCC and 3 AC patients, para-aortic lymph node metastasis was suspected from pre-treatment imaging, and para-aortic lymphadenectomy was therefore also performed. Two patients with stage IB1 SCC underwent radical trachelectomy for preservation of fertility and 1 patient with stage IB1 SCC underwent only conization, at her request. Twenty-nine SCC patients and 4 AC patients were treated with CCRT. Radiotherapy comprised external radiotherapy and brachy-therapy. If lymph node metastasis was suspected from pre-treatment imaging, additional boost irradiation to the lymph nodes was added. Nine SCC patients and 1 AC patient underwent boost irradiation to the pelvic or para-aortic lymph nodes. In SCC, concomitant chemotherapy was administered with a platinum-containing drug; 23 patients received cisplatin (CDDP) and 6 patients received nedaplatin (NDP). In AC, 1 patient received CDDP/NDP, 2 patients received paclitaxel (PTX)/carboplatin (CBDCA), and 1 patient received CDDP/PTX. Five SCC patients and 1 AC patient were treated with radiation alone because of chronic renal dysfunction or old age. Two AC patients received chemotherapy alone.

***FDG-PET/CT scanning***

 PET/CT was performed on two dedicated PET/CT scanners (Discovery ST Elite GE Healthcare, Waukesha, WI, n=62; Discovery IQ, 5-ring detector configuration, GE Healthcare, n=56). After fasting for at least 4 h, all patients received intravenous injection of 105–273 MBq (mean, 132 MBq) of ^18^F-FDG. PET data acquisition started at 62±7 min post-injection, scanning from the top of the skull to the mid-thigh with 2–3 min per bed position. Low-dose non-contrast CT was performed on the same areas. CT data were used for attenuation correction. PET images were reconstructed using an ordered-subset expectation maximization-based algorithm (2 iterations and 14 subsets for Discovery ST Elite; 4 iterations and 12 subsets for Discovery IQ). No harmonization was done between the two scanners.

***Image analysis***

 All PET/CT images were reviewed on a dedicated workstation (Advantage Workstation v.4.6; GE Healthcare). PET/CT images were analyzed quantitatively for primary cancer by one nuclear medicine physician and qualitatively for lymph node (LN) metastasis by two nuclear medicine physicians (one a board-certified nuclear medicine physician). For each scan, SUV_max_, SUV_mean_, MTV, and TLG of the primary cancer were measured. For delineation of the volume of interest (VOI), we referred to previous reports describing a new segmentation algorithm designated as the “estimated threshold method”, with a weighted factor of 0.6 (22, 23). When automatic segmentation included obvious non-tumor parts such as the urinary tract, these areas were manually excluded. SUV_max_ was defined as the highest uptake value within each VOI. MTV was defined as the volume delineated by estimated threshold. SUV_mean_ was defined as the mean SUV within the MTV region. TLG was obtained by the products of MTV and SUV_mean_. LN status was evaluated qualitatively, in which LNs with higher uptake than surrounding background were defined as PET-positive LNs. LN status was divided into three categories: none; pelvic LN only; and pelvic and para-aorta LN. Tumor size was defined as the maximum diameter of the primary tumor measured on T2-weighted MR images. PET/CT and MRI were performed within 30 days of each other.

***Histopathological subtype analysis***

 A variety of subtypes exist in cervical AC, and gastric type mucinous adenocarcinoma (GAC) is a rare variant of mucinous AC. The majority of GAC cases were diagnosed at an advanced stage (59% stages II–IV), while usual-type patients mostly presented at stage I (24). GAC is considered to be associated with poorer prognosis and outcomes compared with usual type (25, 26). Our study included 5 cases of GAC. SUV_max_, SUV_mean_, MTV, and TLG were compared between GAC and other histological types. Using Receiver-operating characteristic (ROC) analyses, we compared the AUC of each PET parameter for OS in all AC cases and the cases excluding GAC.

***Statistical analysis***

 All statistical analyses were performed with JMP version 14.1.0 software (SAS Institute, Cary, NC). Values of p<0.05 were considered statistically significant. Each quantitative PET/CT, MRI, and clinical index was compared between SCC and AC using Mann-Whitney’s U test. Chi-squared test was used to compare the frequencies of categorical variables such as LN status, FIGO stage between SCC and AC. Overall survival (OS) was defined as the time from initial PET/CT to death. Patients alive at the date of last follow-up were censored. ROC analyses were performed separately for SCC and AC to determine optimal cutoffs for SUV_max_, SUV_mean_, MTV, TLG, and tumor size using Youden’s index in terms of developing death. Kaplan-Meier analysis with log-rank testing was used to evaluate differences between two groups divided according to the cutoffs determined from ROC analyses. The prognostic values of imaging parameters and clinical parameters including age, FIGO stage, and type of treatment were assessed using univariate Cox proportional hazard model. Cutoffs for age and FIGO stage were determined from previous report (18). Multivariable analyses were performed among PET/CT parameters and clinical parameters showing p<0.15 in the univariate analysis. Multivariable analysis using both SUV_max_ and SUV_mean_ was not possible, since these values were correlated (correlation coefficient=0.83), so only SUV_max_ was used for multivariable analysis. In addition, since MTV, TLG, and tumor size were correlated (correlation coefficient=0.94 for MTV and TLG, 0.70 for MTV and tumor size, 0.74 for TLG and tumor size), only MTV and TLG were used separately for analysis (MTV in Model 1, TLG in Model 2).

## Results

***Clinical course***

 Median duration of follow-up was 32 months (range, 6–91 months) in SCC and 28 months (range, 6–72 months) in AC. In SCC, at the time of last follow-up, 51 patients (61 %) were alive without recurrence, 23 (28%) had developed locoregional recurrence or metastasis, 12 (14 %) had died from disease progression, and 2 had died from other diseases. Seven (8 %) were lost to follow-up and were censored. In AC, 23 patients (66 %) were alive without recurrence, 10 (29%) had developed locoregional recurrence or metastasis, and 6 (17%) had died from disease progression. Two (6 %) were lost to follow-up and were censored. No significant difference in the rates of death and recurrence was observed between SCC and AC (p=0.71, 0.92, respectively).

***Quantitative indices and LN status in SCC and AC***

**Figure 1:**
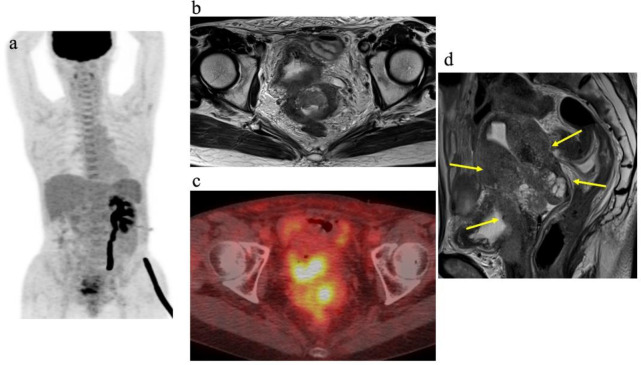
A 54-year-old patient, FIGO stage IVA. (**a**) Maximum-intensity-projection (MIP) PET image, (**b**) axial T2-weighted image, (**c**) axial PET/CT fusion image, and (**d**) sagittal T2-weighted image. Sagittal T2-weighted image shows a tumor with intermediate signal invading from the uterine cervix to the uterine body, vagina, and posterior wall of the bladder (**arrows**). SUV_max_ of the tumor is 6.4 and MTV is 23.5 ml. Left hydronephrosis is observed due to tumor invasion (**a**)

 Values of each quantitative index are presented in Table 2. Representative images from a patient with AC are provided in Figure 1. Mean values of SUV_max_, SUV_mean_, and TLG were significantly higher in SCC than in AC (p<0.01 each). No significant difference in MTV was seen between the two groups (p=0.10). Tumor size was significantly larger in SCC than in AC (p=0.02). In SCC, 19 patients (23%) showed only pelvic lymph nodes, and 12 patients (14%) had both pelvic and para-aorta lymph nodes. In AC, 8 patients (23%) had only pelvic lymph nodes, and 4 patients (11%) had both pelvic and para-aorta lymph nodes. No significant difference in the positive rates of pelvic and para-aorta lymph node was observed between SCC and AC (p=0.75, 0.66, respectively).

**Table 2:** Quantitative PET/CT and MRI indices

	**S** **CC (n=83)**	**A** **C (n=35)**	**p value**
SUV _max_	14.9±6.4	10.5±7.6	<0.01
SUV_mean_	8.0±3.2	6.3±4.7	<0.01
MTV	32.2±37.1	21.5±23.1	0.10
TLG	283.9±345.5	121.6±127.7	<0.01
Size	42.5±16.5mm	35.0±18.6mm	0.02

***ROC analysis***

 From ROC analyses with Youden’s index, optimal cutoffs were determined for quantitative PET/CT indices for predicting OS. 

 In SCC, the cutoff for OS was 14.0, 8.9, 99.8 and 88.7 for SUV_max_, SUV_mean_, MTV and TLG, respectively. In AC, the cutoff for OS was 13.9, 4.5, 32.8 and 173.2 for SUV_max_, SUV_mean_, MTV and 

TLG, respectively. Each cutoff was used for subsequent survival analyses. In SCC, SUV_max_, SUV_mean_, MTV, and TLG showed moderate accuracy (AUC: 0.66, 0.61, 0.61, and 0.63, respectively). In AC, SUV_max_ and SUV_mean _showed low accuracy (AUC: 0.44 and 0.46, respectively), while MTV and TLG showed greater accuracy (AUC: 0.84 and 0.82, respectively).

**Figure:**
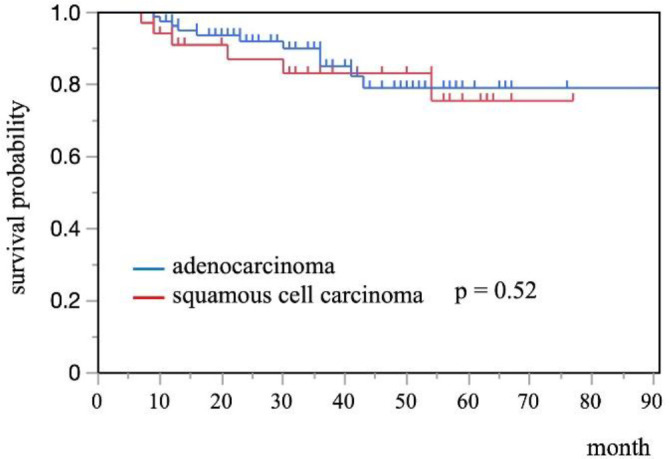
Kaplan-Meier curves of SCC and AC demonstrated that there was no significant difference between SCC and AC for OS (5 year survival rate: 79% vs 75%, p=0.52)

***Survival analysis***

**Figure 3:**
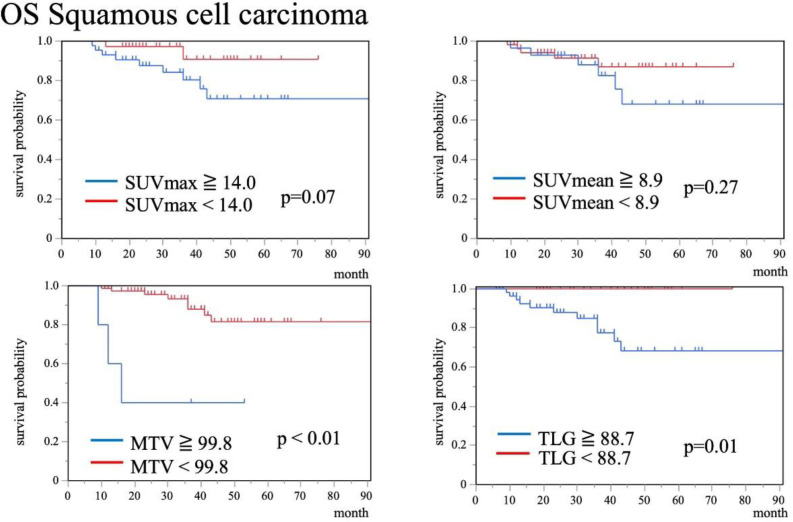
Kaplan-Meier curves of OS for SCC patients stratified by SUV_max_, SUV_mean_, MTV, and TLG. Higher SUV_max_, SUV_mean_, MTV, and TLG tended to show worse OS compared to those with lower values (p=0.07, p=0.27, p<0.01, p=0.01, respectively)

 Kaplan-Meier curves of each histopathological type demonstrated that there was no significant difference between SCC and AC for OS (5 year survival rate: 79% vs 75%, p=0.52) (Figure 2). Kaplan-Meier curves of each PET/CT index for OS are shown in Figure 3, 4. In SCC, patients with SUV_max_, SUV_mean_, MTV, and TLG exceeding cutoff 

values tended to show worse OS than patients with lower values (p=0.07, p=0.27, p<0.01, and p=0.01, respectively). On the other hand, in AC, patients with MTV and TLG exceeding cutoff values showed significantly worse PFS and OS (p<0.01, p<0.01, respectively), while SUV_max_ and SUV_mean_ were unrelated to OS (p=0.91 and p=0.83, respectively).

**Figure 4:**
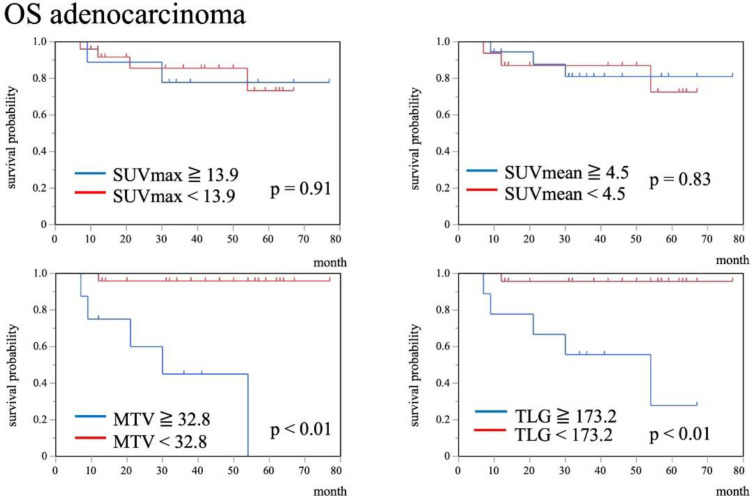
Kaplan-Meier curves of OS for AC patients stratified by SUVmax, SUVmean, MTV, and TLG. Higher MTV and TLG showed significantly worse OS (p<0.01 each), while SUVmax and SUVmean were unrelated to OS (p=0.91, p=0.83, respectively)

 Results of univariate Cox regression analyses are summarized in Table 3. In SCC, MTV, TLG, FIGO stage, and treatment method (surgery or others) correlated significantly with poorer OS (p=0.01, p<0.01, p=0.04, and p=0.05, respectively). 

 In AC, MTV, TLG, tumor size, age, FIGO stage, and treatment method correlated significantly with poorer OS (p<0.01, p<0.01, p<0.01, p=0.03, p=0.02, and p<0.01, respectively).

are shown in Table 4. In SCC, while Model 1 analysis including MTV yielded no significant prognostic factors for OS, analysis including TLG (Model 2) revealed TLG as an independent prognostic factor for both OS (p<0.01). In AC, analysis including MTV (Model 1) showed MTV as an independent prognostic factor for OS (p=0.02), while Model 2 analysis yielded no significant prognostic factors.

 Results of multivariable Cox regression analyses

**Table 3:** Results of univariate Cox regression analyses with OS in SCC and AC

**SCC (n=83)**		
	**O** **S**	
	HR (95%CI)	p value
SUV_max_	3.62 (0.93-23.76)	0.06
SUV_mean_	1.91 (0.57-6.65)	0.29
MTV	7.48 (1.71-27.28)	0.01
TLG	NA	< 0.01
Size	2.39(0.63-7.93)	0.01
Age	1.39(0.40-6.39)	0.62
Lymph node(positive /negative)	1.46 (0.42-4.82)	0.54
Paraaortic lymph node(positive /negative)	1.56 (0.24-6.08)	0.59
FIGO(Ⅲ-ⅣA / I B-Ⅱ)	3.62 (1.05-16.56)	0.04
Treatment(others / surgery)	3.40 (1.02-13.13)	0.05
**AC (n=35)**		
SUV_max_	1.10 (0.15-5.67)	0.91
SUV_mean_	0.84 (0.15-4.56)	0.84
MTV	23.37 (3.64-454.85)	< 0.01
TLG	15.41(2.46-296.17)	< 0.01
Size	11.15 (1.79-213.76)	< 0.01
Age	7.39 (1.17-140.41)	0.03
Lymph node(positive /negative)	1.83 (0.34-9.97)	0.46
Paraaortic lymph node(positive / negative)	2.28 (0.12-14.60)	0.50
FIGO (Ⅲ-ⅣA / I B-Ⅱ)	8.14 (1.30-156.59)	0.02
Treatment (others/ surgery)	24.02 (3.86-460.85)	<0.01

**Table 4:** The results of multivariate Cox regression analyses with OS in SCC and AC

**SCC**					
**O** **S**					
** Model.1**			** Model.2**		
	**HR (95%CI)**	**p value**		**HR**	**p value**
**V** **ariables**			**Variables**		
SUV_max_	1.99 (0.37 – 10.65)	0.40	SUV_max_	1.01 (0.20 – 4.96)	0.98
MTV	3.66 (0.78-17.16)	0.11	TLG	NA	0.01
FIGO	1.81 (0.37-8.76)	0.46	FIGO	1.80 (0.41-7.91)	0.42
Treatment(others/surgery)	1.47 (0.32-6.76)	0.62	Treatment(others/surgery)	1.91 (0.49-7.45)	0.34
**AC**					
**OS**					
** Model.1**			** Model.2**		
	**HR (95%CI)**	**p value**		**H** **R**	**p** ** value**
MTV	45.48(1.69-19775.35)	0.02	TLG	7.31 (0.96-152.37)	0.06
FIGO	NA	0.31	FIGO	NA	0.46
age	7.79 (0.83-256.03)	0.08	age	3.82 (0.54-82.23)	0.20
Treatment(others/surgery)	NA	0.33	Treatment(others/surgery)	NA	0.11

***Histopathological subtype analysis***

 SUV_max_ and SUV_mean_ in GAC tended to be lower than those in ACs other than GAC (6.5 2.3 vs 11.2 7.6, p=0.19 and 3.8 1.0 vs 6.7 4.9, p=0.18, respectively), although not significant. In contrast, MTV in GAC was significantly higher than those of ACs other than GAC (33.7 14.0 ml vs 19.5 23.9 ml, p=0.03). In all AC cases, The AUCs for SUV_max_, SUV_mean_, MTV and TLG for OS were 0.44, 0.46, 0.84 and 0.82, respectively), while in the cases excluding GAC, the AUCs for SUV_max_, SUV_mean_, MTV and TLG for OS were 0.48, 0.48, 0.83 and 0.80, respectively).

## Discussion

 Prognostic stratification is important in deciding on treatment strategies for cervical cancer. If pre-treatment PET/CT could identify patients at high risk of recurrence or resistance to treatment, more personalized treatment options could be provided, such as intensive follow-up or consideration of alternative treatment plans. This study assessed the predictive value of pretreatment PET/CT parameters according to histopathological types of cervical cancer. The present study indicated that in SCC patients, higher quantitative PET/CT indices showed worse OS than those with lower values. In contrast, in AC patients, SUV_max_, and SUV_mean_ were not significant prognostic indicators and only higher MTV and TLG were associated with worsened OS. Multivariable analyses indicated that, in SCC, TLG was an independent prognostic factor for OS. In AC, MTV was an independent prognostic factor for OS.

 As for the prognostic value of PET/CT indices in cervical cancer patients, some reports have demonstrated that pre-treatment SUV_max_ correlated with prognosis (12, 16, 17), while other studies have failed to show any significant association between SUV_max_ and prognosis (27-29). One of the reasons might be that analyses in most studies were performed by mixing SCC and AC, for which the clinical significance of quantitative values would have differed.

 The present study indicated that the prognostic significance of SUV_max_ and SUV_mean_ depended on the histopathological type of cervical cancer, as either SCC or AC. Rahman et al. concluded that SUV_max_ was useful, but MTV was not useful for the prediction of prognosis for non-SCC (18), in addition, Chou et al also indicated that SUV_max_ was related to OS (19). These results differed from the finding in our study, in which MTV and TLG could be good prognostic indices and SUV did not correlate with AC prognosis. This may be partly because the distribution of FIGO stages differed from that in our study. Second, these studies dealt with AC and ASC as a same group, in contrast, present study treated only AC, which may contribute the difference of the result. Third, because of the existence of various subtypes in AC and the differing biological and histological behaviors for each subtype, AC can be regarded as a very heterogeneous carcinoma (2, 3). 

 Therefore, if the distribution of subtypes is different, parameters related to prognosis may differ.

 The presence of para-aorta LN metastasis has long been found to be one of the most significant prognostic factors for cervical cancer patients (30, 31). However, it was not a significant prognostic factor for the cox regression analyses in this study. Khebbeb et al indicated that the sensitivity of PET/CT for para-aorta LN metastasis of cervical cancer was 55% (32). Although in the current study, the exact sensitivity was not revealed due to the lack of histological assessment for para-aorta LN in many cases, it was assumed that the LN status on PET/CT did not accurately reflect the prognosis due to the low sensitivity for para-aorta LN metastasis.

 In this study, SUV_max_ and SUV_mea_n were significantly lower in AC than in SCC. This is consistent with a previous report (33). For lung cancer, AC is also known to show less FDG uptake than SCC (34). The cause of differential uptake remains uncertain, but the involvement of tumor differentiation and tumor micro-environment factors such as inflammatory cells and expression of glucose transporter have been considered (33, 35).

 A variety of subtypes exist in cervical AC, and gastric type mucinous adenocarcinoma (GAC) is a rare variant of mucinous AC. SUV_max_ and SUV_mean_ in GAC tended to be lower than those of other histopathological subtypes, and MTV was significantly higher. GAC grows forming fibrous or edematous desmoplastic stromal reaction without the formation of distinct masses (36, 37), resulting in lower SUV, while the highly infiltrating nature may result in high MTV. These characteristics might have partly caused the differences as a prognostic index of quantitative parameters, however, due to the small number of cases, the contribution to the prognosis was small in the current study.

 Several limitations to this study must be considered. First, the use of two different PET/CT scanners might have contributed to variability in SUV but would not have affected the results substantially. Second, since this was a single institute investigation, some selection bias for patients might have existed. Third, the number of AC patients was small (n=35), and different distributions of histological subtypes in AC might have led to different results.

## Conclusion

 Our preliminary data suggest that PET/CT would be useful for predicting prognosis in cervical cancer, although the clinical significance of quantitative values may differ according to histopathological type.

## Conflicts of interest and sources of funding

 This research did not receive any specific from funding agencies in the public, commercial, or not-for-profit sectors.
